# Determinants of implanon discontinuation among women who ever used implanon in Shashemene district, west Arsi zone, Southern Ethiopia: unmatched case control study

**DOI:** 10.1186/s40834-023-00248-6

**Published:** 2023-10-03

**Authors:** Bikila Lencha, Sintayehu Gabisa Daba, Junayde Abdurahmen Ahmed, Asefa Washo, Girma Beressa, Aster Yalew, Gemechu Ganfure

**Affiliations:** 1 Department of Public Health, Madda Walabu University, Shashemene, Oromia Ethiopia; 2 Center for disease control CDC-Ethiopia, Bishoftu, oromia Ethiopia; 3 Paradise Valley College, Shashemene, Oromia Ethiopia; 4 Department of Public Health, Madda Walabu University, Bale Goba, Oromia, Ethiopia; 5 Department of Nursing, Madda Walabu University, Shashemene, Oromia Ethiopia; 6 Department of pediatrics, Ambo University, Ambo, Oromia Ethiopia

**Keywords:** Implanon discontinuation, Women, Implanon, Ethiopia

## Abstract

**Background:**

Despite tremendous work has been done on demand creation, capacity building and ensuring the logistics of Implanon; its discontinuation rate remained high in Ethiopia; the prevalence is reported to be 31% in Shashemene District. However, the factors contributing to the high prevalence of early Implanon discontinuation were not well understood in our study setting.

**Objective:**

This study aimed to identify the determinants of implanon discontinuation among women who had ever used Implanon in Shashemene District, Southern Ethiopia.

**Methods:**

A community-based unmatched case-control study was conducted among randomly selected 264 women (88 cases and 176 controls) in Shashemene District, Southern Ethiopia, from April 12 to May 18, 2021. A systematic random sampling technique was used to select the respondents. Cases were women who discontinued Implanon before 3 years and controls were those who used implanon for 3 full years. A pre-tested, interviewer-administered structured questionnaire was used to collect data. Bivariable and multivariable binary logistic regression analyses were performed to identify determinants of Implanon discontinuation. An odds ratio (OR) with a 95% confidence interval (CI) was used to estimate the strength of the association, and significance was declared at a P value of less than 0.05.

**Result:**

The mean age of the respondents was 28.23 (± 5.46) years: 27.27 (± 5.38) years for cases and 28.70 (± 5.5) years for controls. Women with no formal education [AOR = 3.09, 95% CI: (1.20, 8.00)], fewer than four children [AOR = 2.47, 95% CI: (1.20, 5.08)], no history of abortion [AOR = 2.84, 95% CI: (1.25, 6.46)], being new acceptors [AOR = 2.14, 95% CI: (1.02, 4. 49)], being counseled for less than fifteen minutes [AOR = 2.47, 95% CI: (1.29, 4.70)], not discussing it with a partner [AOR = 2.88, 95% CI: (1.42, 5.84)] and experiencing side effects [AOR = 0.35, 95% CI: (0.17, 0.71)] were significantly associated with discontinuation of implanon.

**Conclusion:**

Women with no formal education, having less than four children, history of abortion, new acceptors, duration of counseling, discussion with partner, and side effects were determinants of Implanon discontinuation among women. There is a need to ensure adequate pre-implantation counseling and appropriate management of side effects. Furthermore, interventions should target new acceptors and those without formal education.

**Supplementary Information:**

The online version contains supplementary material available at 10.1186/s40834-023-00248-6.

## Introduction

Implanon is a small, soft, flexible, plastic rod, 4 centimeters in length and 2 millimeters in diameter, which containing 68 mg of etonogestrel [1]; and non-biodegradable, subdermal rod approved for up to 3 years of use [2]. It is a long-acting, reversible method of contraception [3] that prevents pregnancy by inhibiting ovulation, causing thickening of the cervical mucus to prevent sperm penetration, and altering the lining of the uterus [4].

Implants are highly effective methods with a pregnancy rate of 0.01–0.1% per year in typical use [5], and the release rate decreases over time from approximately 60–70 g/day in weeks 5–6 to approximately 25–30 µg/day at the end of the third year [6]. Globally, 23 million women were using implants in 2019, representing 2% of all method users [7]. In sub-Saharan Africa, Implanon is underutilized despite its efficacy and low cost [8]. According to the Ethiopian mini-demographic and health survey 2019 report, implant users in the Oromia region were 7.4%, which was lower than the national level of 9% [9].

In 2009, the Ethiopian government launched the Implanon scale-up initiative, which facilitated greater access to Implanon by allowing Health Extension Workers (HEWs) to insert the implant. However, Implanon discontinuation, defined as stopping use of Implanon before three full years, has been the biggest challenge [10]. A few systematic reviews and meta-analyses have been conducted in Ethiopia, both of which reported a prevalence of Implanon discontinuation of more than 32% [11, 12]. However, they suffered from very high heterogeneity of more than 97% [11, 12]. This is due to variations in the population and the definition of ID. Evidence from the 2016 Ethiopian Demographic and Health Survey (EDHS) shows that 21.5% of implant episodes were discontinued at the end of 24 months, before reaching the intended duration of use [13]. Studies from different parts of Ethiopia also show that implanon discontinuation remains high, ranging from 16% in Tigray Region [14] to 65% in Debre Tabor City [15]. The most recent review from Ethiopia also reported that the pooled prevalence of implant discontinuation was 36.95% [16].

Different studies conducted on Implanon discontinuation have shown that socioeconomic and demographic factors (age, education level of women and partners, marital status, occupation, income, place of residence), counseling-related factors (pre-insertion counseling, follow-up, satisfaction with service), obstetric factors (number of children, abortion, desire to become pregnant), and method-related factors (side effects, health concerns) were associated with Implanon discontinuation [14, 15, 17–27].

Evidence shows that contraceptive discontinuation varies by type of contraceptive used, age, race/ethnicity, and when contraceptives were first used [28–30]. Since there are socio-cultural variations in the different regions of Ethiopia; there are also variations in the determinants of implanon discontinuation in different settings among these diverse groups. Identifying the factors that would lead to discontinuation of implanon use will ensure better use and thus help to increase the continuation rate of implanon users in preventing unintended pregnancy and improve future appropriate implanon services in West Arsi Zone in general and Shashamane district in particular. The study would help the health professionals and the District Health Office to take appropriate measures to address the major factors leading to the discontinuation of implanon. Therefore, this study aimed to identify the determinants of Implanon discontinuation among women who had ever used Implanon in Shashemene District, Southern Ethiopia.

## Methods

### Study design and period

A community-based, unmatched case-control study was conducted in Oromia Regional State, West Arsi Zone, Shashemene District from April 12 to May 18, 2021. It is located 250 km south of the capital city of Ethiopia, Addis Ababa. Shashemene District has 281,247 population, 58,593 households and 61,874 women of reproductive age group living in 37 rural kebeles (small administrative units in Ethiopia). There are 7 health centers and 38 health posts providing health services to the community through 91 health extension workers and 144 health workers. In addition, there were 11 private clinics. All health centers and health posts provide implant insertion and removal services. The prevalence of long-acting family planning use according to the annual report in 2020/2021 was 17.4% and 26.8% in West Arsi Zone and Shashemene District, respectively. Similarly, according to this report, among women who have used implanon in 2020/21; 23% of them in West Arsi Zone and 31% of them in Shashemene District have discontinued before the intended time [31].

### Study participants

All women of childbearing age who had ever used an implanon in Shashemene District were the source population for this study.

#### Cases

All women of child bearing age in the selected kebeles who discontinued their implanon before 3 years of insertion and were registered from January 1, 2018 to January 1, 2021.

#### Controls

All women of reproductive age who removed their implanon after 3 full years of use and who were registered from January 1, 2018 to January 1, 2021.

### Inclusion and exclusion criteria

Women of reproductive age who were eligible for cases (discontinued Implanon before 3 years) and controls (used Implanon for the full 3 years) and who resided in the study district were included in the study.

Women of reproductive age who discontinued Implanon because of life-threatening medical complications or method failure and who were critically ill at the time of the interview were excluded from the study.

### Sample size determination and sampling Procedure

The sample size was calculated using two population proportion formula for an unmatched case-control study from Epi info version 7.2.2 with the assumptions of a significance level of 5%, power of 80%, control to case ratio (2:1), odd ratio of 2.3, and percentage of controls with a history of abortion, 28.6% [17], and a non-response rate of 10%. The final sample size was estimated to be 264 (88 cases and 176 controls) (Table 1).

**Table 1:** Sample Size Determination

Main factors	CI	Power	Ratio	% CWE	OR	Cases	Controls	Total	References
Discussion a with partner	95%	80	1:2	23.4	2.87	54	107	161	[32]
Side effects	95%	80	1:2	56.5	2.66	66	132	198	[33]
Previous history of abortion	95%	80	1:2	28.6	2.3	88	176	264	[17]

CI: Confidence Interval OR: Odds Ratio % CWE: Percent of control with exposure

There were 37 kebeles (small administrative units) in Shashemene District, and one third of the District kebeles (thirteen kebeles) were selected by lottery. The lists of women were obtained, and a sampling frame was prepared from implanon insertion and removal registration book of health posts and health centres (totally 810, including 281 cases and 529 controls). The proportional allocation was done to determine the number of women to be included from 13 Kebeles selected. A systematic random sampling method was applied to select study participants. Every third women was selected for both cases and controls. If there is no eligible woman in selected household, the next household would be approached for the study.

### Data collection tool and procedure

The study used a pre-tested, structured, interviewer-administered questionnaire adapted from various literatures for data collection [14, 17, 32, 34]. The questionnaire contained: socio-economic and demographic characteristics of mothers; obstetric history; past knowledge and utilization of contraceptive methods; counseling related factors and reasons for removal of implanon (S1). Data collection was carried out by going home to home in selected kebeles of Shashemene District by five diploma nurses as data collectors and two-degree holding nurses as supervisors. The data were collected from women of child bearing age group by face-to-face interview.

### Variable measurement

Implanon discontinuation was defined as the discontinuation of the use of implanon before completion of three years [10]. Card of mothers were used to check the date of insertion and removal of implanon. In the absence of a card, women’s self-reports were cross-checked with their files from the health post or health centres.

Independent variables were categorised as follows; age (< 20, 20–24, 25–29, 30–34 & ≥ 35 years), place of residence (rural, semi-urban), marital status (married & others), ethnic group (Oromo, Sidama & others), religion (Muslim, Orthodox & Protestant), women occupation (housewife, farmer & others), partner occupation (farmer, merchant & others), women education(unable to read & write, able to read and write, primary & secondary), partner education (unable to read & write, able to read and write, primary, secondary, college & above), have children before insertion(yes, no), number of children (1–3, 4 and above), future intention to have children (yes, no), history of abortion (yes, no), ever used contraceptive before Implanon(yes, no), type of counselling (individual, with husband, mass), duration of counselling (< 15 min & ≥ 15 min), discussed with partner to use Implanon (yes, no), decision maker to use Implanon (self, husband & other), Implanon provider (health extension worker & health worker), side effect after insertion (yes, no), follow-up after insertion (yes, no), service satisfaction(yes, no) and unintended pregnancy after removal of Implanon (yes, no). Semi-urban was defined as the newly established residential areas within a one-kilometre radius of the urban areas. A woman is considered to have experienced side effects from implanon if she reports one of the following conditions: menstrual disruption, headache, weight gain, insertion arm pain, insertion site infection, back pain, or expulsion.

### Data quality control

The questionnaire was prepared in an English version, translated into the local language (Afaan Oromoo) and then back translated to English to check its consistency. Before data collection, one day of training was given for data collectors and supervisors about the purpose of the study, data collection tools and ethical issues. The questionnaire was pre tested on 5% (4 cases and 8 controls) of the calculated sample size in nearby kebele (Abaro Kebele) before the actual data collection period. The result of the pretest was used to make amendments before carrying out the study.

The collected data were checked daily by the supervisors for completeness, consistency, and cleanness and investigators monitored the overall quality of data collection. Participants were requested to give honest responses during the interview. Any error found during the interview was corrected immediately. All the results were reported as per the recommendations of the STROBE guideline (S2).

### Data processing and analysis

The data were checked for completeness and entered into Epi Info version 7.2.2 and imported into the Statistical Package for Social Science (SPSS) version 25.0 for analysis. Descriptive statistics, including frequency, proportion, mean and standard deviations were computed to describe the data. A bivariable and multivariable binary logistic regression analysis was conducted to assess the association between outcome and explanatory variables. Variables that had a P-value of 0.2 in the bivariable logistic regression analyses were entered into the multivariable logistic regression model to control potential confounding effects. Finally, multivariable binary logistic regression analysis was conducted to identify determinants of implanon discontinuation. During multivariable logistic regression analysis, model fitness was checked using Hosmer and Lemeshow`s goodness of fit test (p = 0.412). Multi-collinearity between independent variables was checked using variance inflation factor (VIF) and all variable had VIF less than 2. Adjusted odds ratios (AOR), along with a 95% confidence interval (CI), were used to assess the strength of the association between predictors and the outcome variable. The level of statistical significance was declared at a p-value less than 0.05.

## Results

### Socio demographic characteristics of the participants

A total of 264 women (88 cases and 176 controls) participated in the study, resulting in a 100% response rate for both cases and controls. The mean age of the respondents was 28.23 (± 5.46) years: 27.27 (± 5.39) years for cases and 28.70 (5.53) years for controls. Most of the women, 83 (94.3%) of the cases and 173 (98.3%) of the controls, were married. About one third of the 30 (34.1%) cases and 69 (39.2%) controls had completed primary school. Regarding the occupational status of the partner, most of the 66 (75.0) cases and 136 (77.3) controls partners were farmers (Table 2).

**Table 2:** Socio-demographic characteristics of women who had ever used Implanon in Shashemene Distrcit, Southern Ethiopia, 2021

Variables	Categories	Cases (n = 88)	Control (n = 176)
		n (%)	n (%)
Age	< 20	12 (13.6)	11 (6.3)
	20–24	15 (17.1)	27 (15.3)
	25–29	28 (31.8)	63 (35.8)
	30–34	22 (25.0)	48 (27.3)
	>=35	11 (12.5)	27 (15.3)
Place of Residence	Rural	80 (90.9)	165 (93.8)
	Semi-urban	8 (9.1)	11 (6.3)
Marital status	Married	83 (94.3)	173 (98.3)
	Others *	5 (5.7)	3 (2.7)
Ethnic Group	Oromo	81 (92.0)	159 (90.3)
	Sidama	4 (4.6)	10 (5.7)
	Others**	3 (3.4)	7 (4.0)
Religion	Muslim	80 (90.9)	158 (89.8)
	Orthodox	3 (3.4)	9 (5.1)
	Protestant	5 (5.7)	9 (5.1)
Women`s Occupational status	House wife	80 (90.9)	151 (85.8)
	Farmer	2 (2.3)	17 (9.7)
	Others***	6 (6.8)	2 (1.1)
Partner`s Occupation	Farmer	66 (75.0)	136 (77.3)
	Merchant	16 (18.2)	35 (19.9)
	Others****	6 (6.8)	5 (2.8)
Maternal Educational status	Can`t read and write	27 (30.7)	34 (19.3)
	Read and write	15 (17)	27 (15.3)
	Primary	30 (34.1)	69 (39.2)
	Secondary	16 (18.2)	46 (26.1)
Partner`s Educational level	Can`t read and write	18 (20.5)	13 (7.4)
	Read and write	37 (42.0)	89 (50.6)
	Primary	20 (22.7)	48 (27.3)
	Secondary and above	11 (12.5)	23 (13.1)
	College and above	2 (2.3)	3 (1.7)

* Single, Widowed, ** Amhara, Wolaita, *** Merchant, Student, ****Government employee, daily labourers

### Obstetric history of study participants

This study found that 81 (92.0%) cases and 167 (94.9%) controls had children prior to implantation. This study also showed that 45 (51.1%) cases and 71 (40.3%) controls had 1–3 children. The majority of respondents, 82 (93.2%) cases and 161 (91.5%) controls, had an intention to have children in the future. Of the total study participants, 125 (71.0%) cases and 75 (85.2%) controls had no history of abortion.

### Awareness and use of contraceptive methods

According to the results of this study, the majority of respondents, 75 (85.2%) cases and 160 (90.9%) controls, received information about contraceptive methods from health extension workers. Most of the respondents, 54 (61.4%) cases and 141 (80.1%) controls, had used other contraceptive methods before using implanon. The most common contraceptive method ever used before Implanon was the injectable method: 34 (38.6%) cases and 93 (52.8%) controls. (Table 3).

**Table 3:** Knowledge and use of contraceptive among women who had ever used Implanon in Shashemene District, Southern Ethiopia, 2021

Variables		Categories	Cases(n = 88)	Control (n = 176)
			N (%)	N (%)
Information source		Health Extension Workers	75 (85.2)	160 (90.9)
		Health Workers	9 (10.2)	13 (7.4)
		Others*	4 (4.6)	3 (1.7)
Type of information obtained about contraceptives	Effectiveness	No	47 (53.4)	91 (51.7)
		Yes	41 (46.6)	85 (48.3)
	Side effects	No	49 (55.7)	93 (52.8)
		Yes	39 (44.3)	83 (47.2)
	Duration of action	Yes	70 (79.5)	129 (73.3)
		No	18 (20.5)	47 (26.7)
	Benefits	Yes	63 (71.6)	119 (67.6)
		No	25 (28.4)	57 (32.4)
Ever used contraceptive before		Yes	54(61.4)	141 (80.1)
		No	34(38.6)	35 (19.9)
Last method used before insertion of implanon		Injection	34(38.6)	93 (52.8)
		Pills	20(22.7)	47 (26.7)
		Other**	0	1 (0.6)
Reason for not utilizing any method before insertion		Fear of side effects	25 (28.4)	22 (12.5)
		Need more children	6 (6.8)	11 (6.3)
		Others***	3 (3.4)	1 (0.6)

*Friends, Media Television/Radio, **Intrauterine contraceptive device, *** Marital dissolution, Husband opposed

### Counseling related characteristics of participants

About 70 (79.5%) cases and 140 (79.5%) controls received individual pre-counseling, and 60 (68.2%) cases and 83 (47.2%) controls received counseling for less than fifteen minutes. Among those who used implanon, 54 (61.4%) cases and 140 (79.5%) controls had discussions with their partners. The majority of women, 78 (88.5%) of cases and 147 (83.5%) of controls, received implanon insertion services from health workers. According to this study, 59 (67%) of the cases and 54 (30.7%) of the controls experienced a side effect, and most of the respondents (59 (67%) of the cases and 137 (70.8%) of the controls) were followed up after implantation. Of the 88 cases who discontinued implanon before 3 years, 14 (15.9%) reported that their pregnancy was unintended (Table 4).

**Table 4:** Counseling related characteristics of women who had ever used Implanon in Shashemene District, Southern Ethiopia, 2021

Variables		Categories	Cases(n = 88)	Control (n = 176)
			N (%)	N (%)
Types of Counseling		Individual	70 (79.5)	140 (79.5)
		With husband	9 (10.2)	20 (11.4)
		Mass	9 (10.2)	16 (9.1)
Duration of counseling		< 15 min	60 (68.2)	83 (47.2)
		≥ 15 min	28 (31.8)	93 (52.8)
Information obtained during counseling	About advantage	Yes	82 (93.2)	169 (96)
		No	6 (6.8)	7 (4.0)
	About side effects	Yes	75 (85.2)	153 (86.9)
		No	13 (14.8)	23 (13.1)
	When to insert and remove	Yes	81 (92.0)	164 (93.2)
		No	7 (8.0)	12 (6.8)
	Duration of action	Yes	64 (72.7)	131 (74.4)
		No	24 (27.3)	45 (25.6)
	Effectiveness	Yes	56 (63.6)	100 (56.8)
		No	32 (36.4)	76 (43.2)
	Presence of alternative	No	67 (76.1)	137 (77.8)
		Yes	21 (23.9)	39 (22.2)
Discussed with partner to use implanon		No	34 (38.65)	36 (20.5)
		Yes	54 (61.4)	140 (79.5)
Decision maker to use implanon		Her-self	79 (89.8)	173 (98.3)
		Husband	9 (10.2)	3 (1.7)
Implanon provided by whom?		HEW	78 (88.6)	147 (83.5)
		Health Worker	10 (11.4)	29 (16.5)
Experienced side effects after insertion		No	59 (67)	54 (30.7)
		Yes	29 (33.0)	122 (69.3)
Menstrual cycle pattern after insertion		Regularly	15 (17.0)	73 (41.5)
		Irregular (unscheduled)	39 (44.3)	25 (14.2)
		It already stops	34 (38.6)	78 (44.3)
Follow-up after insertion		No	29 (33.0)	39 (22.2)
		Yes	59 (67.0)	137 (77.8)
Satisfied by service given		No	6 (6.8)	3 (1.7)
		Yes	82 (93.2)	173 (98.3)

* Service provider, HEW – Health extension worker, Health worker:- physicians, health officers, nurses and midwives who provided implanon insertion

### Determinants of implanon discontinuation

In bivariable binary logistic regression, age, maternal education, number of children, history of abortion, previous contraceptive use, duration of counseling, discussion with partner, side effects, menstrual bleeding pattern after insertion, and follow-up appointment were found to be candidate variables for multivariable logistic regression analysis with a p-value < 0.2.

After adjusting for variables in a multivariable logistic regression model, women with no formal education [AOR = 3.09, 95% CI: (1.20, 8.00)], fewer than four children [AOR = 2.47, 95% CI: (1.20, 5.08)], no history of abortion [AOR = 2.84, 95% CI: (1.25, 6.46)], being a new acceptor [AOR = 2.14, 95% CI: (1. 02, 4.49)], being counseled for less than fifteen minutes [AOR = 2.47, 95% CI: (1.29, 4.70)], not discussing it with a partner [AOR = 2.88, 95% CI: (1.42–5.84)], and experiencing side effects [AOR = 0.35, 95% CI: (0.17, 0.71)] were significantly associated with Implanon discontinuation among women.

Women with no formal education were 3.09 times likely to discontinue implanon compared to women with a secondary education [AOR = 3.09, 95% CI: (1.20- 8.00)]. The study also showed that women who had 1–3 living children were 2.47 times likely to discontinue implanon compared to women who had four or more children at the time of implantation [AOR = 2.47, 95% CI: (1.20–5.08)]. The odds of implanon discontinuation were 2.84 times higher in women who had no history of abortion compared to their counterparts [AOR = 2.84, 95% CI: (1.25–6.46)]. The odds of Implanon discontinuation were 2.14 times higher among new users compared to those who had ever used any contraceptive method [AOR = 2.14, 95% CI: (1.02–4.49)]. The analysis showed that women who received less than fifteen minutes of counseling were 2.47 times likely to discontinue Implanon compared to those who received fifteen minutes or more of counseling [AOR = 2.47, 95% CI: (1.29–4.70)]. The odds of Implanon discontinuation were 2.88 times higher among women who had not discussed it with their partner than among those who had discussed it with their partner [AOR = 2.88, 95% CI: (1.42–5.84)]. This study also showed that women who did not experience any side effects after Implanon implantation were 65% less likely to discontinue Implanon use compared to women who experienced side effects after Implanon implantation [AOR = 0.35, 95% CI: (0.17–0.71)] (Table 5).

**Table 5:** Determinant of Implanon discontinuation among women who had ever used Implanon in Shashemene District, Southern Ethiopia, 2021

Variables	Categories	Cases N (%)	Controls N (%)	COR[95%CI]	AOR [95%CI]
Age	< 20	12 (13.6)	11 (6.3)	2.68 (0.91–7.86)*	1.95 (0.36–10.61)
	20–24	15 (17.0)	27 (15.3)	1.36 (0.53–3.50)	1.17 (0.26–5.30)
	25–29	28 (31.8)	63 (35.8)	1.09 (0.48–2.50)	1.31 (0.41–4.21)
	30–34	22 (25.0)	48 (27.3)	1.12 (0.47–2.67)	1.54 (0.52–4.53)
	>=35	11 (12.5)	27 (15.3)	1	1
Maternal Educational status	No formal education	27 (30.7)	34 (19.3)	2.28 (1.07–4.89)*	3.09 (1.20-8.00)**
	Read and write	15 (17)	27 (15.3)	1.60 (0.68–3.74)	2.28 (0.79–6.54)
	Primary	30 (34.1)	69 (39.2)	1.25 (0.61–2.55)	1.39 (0.59–3.27)
	Secondary	16 (18.2)	46 (26.1)	1	1
Number of children	< 4	45 (51.1)	71 (40.3)	1.55 (0.92–2.59)*	2.47 (1.20–5.08)**
	>=4	43 (48.9)	105(59.7)	1	1
History of abortion	No	69 (78.4)	162 (92)	2.35 (1.20–4.61)*	2.84 (1.25–6.46)**
	Yes	19 (21.6)	14 (8.0)	1	1
Ever used contraceptive	No	34 (38.6)	35 (19.9)	2.54 (1.44–4.47)*	2.14 (1.02–4.49)**
	Yes	54 (61.4)	141 (80.1)		1
Duration of counseling	< 15 min	60 (68.2)	83 (47.2)	2.40 (1.40–4.11)*	2.47 (1.29–4.70)**
	>= 15 min	28 (31.8)	93 (52.8)		1
Discussed with partner to use	No	34 (38.6)	36 (20.5)	2.45 (1.39–4.30)	2.88 (1.42–5.84)**
	Yes	54 (61.4)	140 (79.5)		1
Side effects after insertion	No	29 (33)	122 (69.3)	0.22 (0.13–0.38)*	0.35 (0.17–0.71)**
	Yes	59 (67)	54 (30.7)	1	1
Menstrual bleeding pattern after insertion	Regularly	15 (17.0)	73 (41.5)	0.47 (0.24–0.94)*	0.46 (0.21–1.03)
	Irregular(unscheduled)	39 (44.3)	25 (14.2)	3.58 (1.88–6.81)	2.25 (0.99–5.07)
	It already stops	34 (38.6)	78 (44.3)	1	1
Follow-up appointment	No	29 (33)	39 (22.2)	1.73 (0.98–3.05)*	1.11 (0.54–2.27)
	Yes	59 (67)	137(78.8)	1	1

**statistically significant at p < 0.05 in multivariable binary logistic regression analysis; 1 = reference category; AOR = Adjusted Odds Ratio; COR = Crude Odds Ratio

## Discussion

Implanon discontinuation is a major public health problem in low-income countries, including Ethiopia, because it leads to unintended pregnancy. The current study aimed to identify the determinants of ID among women who had ever used Implanon in Shashemene District. Our findings suggest that low level of education, inadequate counseling, having less than four living children, being a new acceptor, and poor communication between couples are important factors that require immediate intervention. Appropriate management of side effects is also very important to avoid implant discontinuation.

This study found that women’s educational status was significantly associated with Implanon discontinuation. This finding was supported by studies conducted in the Wolayta Zone [17], Mekelle City [33], and Ilorin, Nigeria, which strongly agreed that uptake and continuation of Implanon was influenced by the educational attainment [35]. Women with higher level of education continued to use implanon because they may have better awareness compared to uneducated women.

The other determinant associated with ID was having 1–3 living children.This finding was supported by studies conducted done in Duguna Fango District [17] and Agarfa District [36]. The reason may be that women may need to have more children to achieve their desired number of children; thus, they might prefer to discontinue implanon. There is also the possibility of pressure from husbands in need of more children.

The odds of ID were 2.84 times higher among women who had no history of abortion when compared to their counterparts. This study’s finding was supported by the study conducted in Andabet District [37]. This may be due to the fact that women who have ever had an abortion are more likely to need to space their pregnancies using an implanon for the appropriate amount of time than women who have never had an abortion. It might also be due to knowledge acquired after post abortion care and fear of recurrence of abortion. However, this finding is different from the study done in Duguna fango District, Wolayta zone, where women who had previous history of abortion were 2.3 times more likely to discontinue their implanon when compared to those women had not experienced abortion [17]. This might be due to socio-demographic differences between two study settings and needs further qualitative studies.

According to this study, the odds implanon discontinuation among new acceptors was twofold compared to those who had ever used any contraceptive. This finding was supported by the study done in the Kucha District of Gamo gofa Zone [23] .This might be due to repeated exposure to contraceptive use,which has helped them to acquire knowledge about contraceptives. Similarly, the intensity of counseling obtained may vary between the two groups.

This study also showed that women who had counseled for less than fifteen minutes were 2.47 times more likely to discontinue their implanon use when compared to those who had counseled for fifteen minutes and more. This finding implies the importance of comprehensive counseling to ensure that clients are fully informed about the use of the method, including potential side effects. Women who did not discuss it with their partners were also 2.88 times more likely to discontinue implanon as compared to those who did. This is in agreement with studies conducted in Bahir Dar Town [32], Kucha District Gamo Gofa Zone [23], and Bale Zone [36] This might be due to the support of male partners for their spouses to continue utilization of Implanon. In Ethiopian culture, males are predominant decision-makers, and their involvement is very important for the success of health service use, including contraception.

Presence of side effects was associated with Implanon discontinuation. Women who had no side effects after insertion of an implanon were 65% less likely to discontinue their implanon use as compared to women who had experienced side effects after insertion of an implanon. This result is in line with the study conducted in Buffalo City, South Africa [38], Kinshasa [27], Upper Egypt [39], Ofla District [14], Debre Markos [40], Andabet District [37], Mekelle town [33], and Ambo Town [22]. This might be due to a lack of information about possible side effects during pre-insertion counseling by providers. Women’s may be frightened by unanticipated changes in their mood, menstruation patterns, or weight.

### Limitation of the study

Since data collection method was face-to-face interview, there might be possibility of social desirability bias. Social desirability was also possible because of self-reporting of the time period of ID. The possibility was minimized by cross-checking with the family card of the mother and with the health facility. Selection bias was minimized since it was a community based study. Both cases and controls came from the same source population. Because of the nature of the design, there might be recall bias. To reduce recall bias, target women who had discontinued implanon in the three years prior to the data collection were used. It is very difficult to establish cause –effect relationship between the exposures and outcome because of the nature of the design, and the observed associations should be interpreted throughout the manuscript cautiously. There might be possibility of interviewer bias because the interviewers already knew the cases and controls and could ask them differently. Despite the limitations listed above, this study provides valuable evidence about determinants of Implanon discontinuation that could be utilized by programme planners and policy makers to further strengthen the implant use among women in Shashemene, in particular, and in Ethiopia, in general .

## Conclusion

This study indicated that factors influencing women’s decision to discontinue using an implanon were lack of formal education, having less than four children, a history of abortion, being a new user, short counseling time, lack of conversation with a spouse, and presence of side effects. Public health interventions should target partners, new acceptors, and those without formal education. Women’s education and empowerment should be promoted in collaboration with education bureaus and women’s affairs offices. Family planning providers should provide comprehensive counseling to women before providing Implanon. Family planning providers should encourage discussion between couples. Family planning programs should focus on managing the side effects of implanon to reduce discontinuation. Qualitative research should be conducted to further explore barriers to and facilitators of implanon use.

### Electronic supplementary material

Below is the link to the electronic supplementary material.

Supplementary Material 1

Supplementary Material 2

Supplementary Material 3

## Data Availability

The necessary data and material of this study are available from the corresponding author whenever needed.

## Abbreviations

AOR: Adjusted Odds Ratio
CI: Confidence Interval
COR: Crude Odds Ratio
EMDHS: Ethiopian Mini-Demographic and Health Survey
FMOH: Federal Ministry of Health
LARC: Long-acting Reversible Contraceptive Methods
ORHB: Oromia Regional Health Bureau
Pv: P-value
SPSS: Statistical Package for Social Science
WHO: world Health Organization
