# Supplementary material for: Determinants of implanon discontinuation among women who ever used implanon in Shashemene district, west Arsi zone, Southern Ethiopia: unmatched case control study
Source: Contracept Reprod Med. 2023 Oct 3;8:46. doi: 10.1186/s40834-023-00248-6 (PMC10548583; doi:10.1186/s40834-023-00248-6)
Supplement: Supplementary file 1 — Supplementary Material 1 [file 40834_2023_248_MOESM1_ESM.docx]

### **English Version Questionnaires**

**A study on Determinants of implanon discontinuation among women who ever used implanon in Shashamane District, West Arsi Zone, Southern Ethiopia,2020/21**

Name of Kebele ______________ Date of interview: _______________

Time interview started: hour____ minute_____ Time interview ended: hour____ minute ______

Name of Interviewer: _________________ Name of Supervisor: _______________________

**Instruction: -** Encircle the responses for questions with alternatives and for open ended questions write on the space provided.

| **Part I: Socioeconomic and Demographic characteristics** | | | | | |
| --- | --- | --- | --- | --- | --- |
| **S.NO.** | | **Questions** | **Response** | **Skip to/Remark** | |
| 101 | | What is your age in full years? | Age _____ in years |  | |
| 102 | | Place of residence | 1. Rural 2. Semi-urban 3. Urban |  | |
| 103 | | What is your marital status? | 1. Single 2. Married 3. Widowed 4. Divorced/separated |  | |
| 104 | | What ethnic group do you belong to? | 1. Oromo 2. Sidama 3. Amhara 4. Wolaita 5. Others(specify) ------------- |  | |
| 105 | | What is your religion? | 1. Muslim 2. Orthodox 3. Protestant 4. Catholic 5. Others(specify) ------------- |  | |
| 106 | | What is your occupation? | 1. Housewife 2. Farmer 3. Merchant 4. Private Organization employee 5. Government employee 6. Daily laborer 7. Other (specify) ------------- |  | |
| 107 | | What is your husband occupation? | 1. Farmer 2. Merchant 3. Private Organization employee 4. Government employee 5. Daily laborer 6. Other (specify)--------------- |  | |
| 108 | | How many children do you have? | _____________________ |  | |
| 109 | | What is your educational level? | 1. Can`t read and write 2. Read and write 3. Primary 4. Secondary 5. Collage and above |  | |
| 110 | | What is your husband education level? | 1. Can`t read and write 2. Read and write 3. Primary 4. Secondary 5. Collage and above |  | |
| 111 | | What type of House do you have? | 1. Thatched 2. Corrugated Iron Sheet 3. Other (Specify) |  | |
| 112 | | Type of house floor | 1. Earth /Soil/. 2. Cement/Brick 3. Wooden/Bamboo 4. Ceramic 5. Other (Specify) |  | |
| 113 | | How many rooms used by the member of your household for sleeping? | ______________ rooms |  | |
| 114 | | Do you have livestock, herd or farm animal? | 1. Yes  2. No | If no, skip to 116 | |
| 115 | | If yes, how many? | Number |  | |
|  |  | Chickens |  |  | |
|  |  | Sheep |  |  | |
|  |  | Goat |  |  | |
|  |  | Cows |  |  | |
|  |  | Ox |  |  | |
|  |  | Donkey |  |  | |
|  |  | Horse |  |  | |
| 116 | | How many agricultural lands do you have? | 1. Do not have 2. Acres _____ (local unit Qarxi ____) 3. Do not know/not sure |  | |
| **Part II: Obstetric History** | | | | | |
| **S.NO.** | | **Questions** | **Response** | **Skip to/Remark** | |
| 201 | | Did you have children during insertion of implanon | 1. Yes 2. No | If No skip to 203 | |
| 202 | | If yes how many children did you have? | ______________ |  | |
| 203 | | Do you intend to have children? | 1. Yes 2. No | If No skip to 205 | |
| 204 | | If yes how many do you want to have? | ______________ |  | |
| 205 | | Did you have any history of abortion? | 1. Yes 2. No | If No skip to 301 | |
| 206 | | If yes, how many times? | ______________ |  | |
| **Part III: Past knowledge and utilization of contraceptive methods** | | | | | |
| **S.NO.** | | **Questions** | **Response** | | **Skip to/Remark** |
| 301 | | Have you ever heard of any contraceptive methods? | 1. Yes 2. No | | If No skip to 305 |
| 302 | | Which kind of contraceptive method have you ever heard of? You can answer more than one. | 1. Implanon 2. Jadelle 3. IUCD 4. Injectable 5. Pills 6. Female Sterilization 7. Male Sterilization 8. Male condom 9. Others (specify)………… | |  |
| 303 | | From where did you obtain the information? | 1. Friends 2. HEW 3. Health workers 4. Media/TV/Radio 5. Others/specify__________ | |  |
| 304 | | What type of information did you know? (Multiple answers possible) | 1. Effectiveness 2. Side effects 3. Duration of action 4. Benefit 5. Others (specify) ………… | |  |
| 305 | | Have you ever used any contraception before using Implanon? | 1. Yes 2. No | | If No skip to 307 |
| 306 | | Which method you did use? (last method) | 1. Pills 2. IUCD 3. Injectable 4. Others (specify)………… | |  |
| 307 | | What was the main reason for not using any contraceptive method before? | 1. Fear of side effects 2. Need more children 3. Husband away 4. Marital dissolution 5. Husband opposed 6. Others/specify__________ | |  |
| **Part IV: Counseling related factors** | | | | | |
| **S.NO.** | | **Questions** | **Response** | | **Skip to/Remark** |
| 401 | | Did you get counseling service before inserting the implanon? | 1. Yes 2. No | | If No skip to 405 |
| 402 | | What type of counseling did you obtain? | 1. Individual counseling 2. Mass counseling 3. With husband counseling 4. Other/specify _________ | |  |
| 403 | | For how long did you get counseling? | 1. For < 15 minutes 2. For ≥ 15 minutes | |  |
| 404 | | What type of information did you obtain during the counseling? (Multiple answers possible) | 1. Advantage 2. Side effects 3. Duration of action 4. Effectiveness 5. When to insert and remove 6. The presence of alternatives 7. Other (specify) | |  |
| 405 | | Did you first discuss with your partner exactly to use this method of FP? | 1. Yes 2. No | | If yes, skip to 407 |
| 406 | | Why not you discuss with your husband? | 1. Husband opposes 2. Husband away 3. Alone at that time 4. Other | |  |
| 407 | | Did he accept at that time? | 1. Yes 2. No | |  |
| 408 | | With whom did you go to the Clinic? | 1. With husband 2. Alone 3. Other /specify …………. | |  |
| 409 | | Who was decided inserting the Implanon | 1. Husband 2. My self 3. Service provider 4. Other /specify ----------- | |  |
| 410 | | Where did you insert Implanon? | 1. Health post 2. Health center 3. Hospital 4. Private clinic 5. Other /Specify---------- | |  |
| 411 | | Who did insert Implanon for you? | 1. HEW 2. HW 3. Other/ specify--------- | |  |
| 412 | | Why did you choose to use Implanon? | 1. I satisfied with it 2. It is convenient to me 3. Most of my friends using it 4. Provider initiated me to take it 5. I myself wanted to take it 6. My husband wanted me to take it 7. Exposure to positive media 8. Other(specify) --------------- | |  |
| 413 | | How long did it take to reach the source of implanon from your home? | _________ KM | |  |
| 414 | | Did you feel any side effect after inserting Implanon? | 1. Yes 2. No | | If No skip to 416 |
| 415 | | If yes, what type of side effect(s)? | 1. Insertion site pain 2. Infection on insertion site 3. Expulsion 4. Heavy/prolonged bleeding 5. Headache 6. Weight gain 7. Others/specify---------- | |  |
| 416 | | How it comes your menstrual cycle after you have started using implanon? | 1. Regularly 2. Irregular(unscheduled) 3. It already stops | | If already stops skip to 418 |
| 417 | | Was there any change in the amount of menstrual loss after you have started using Implanon? | 1. No change 2. Increased 3. Decreased 4. Others (specify | |  |
| 418 | | After the insertion of implanon, did FP service provider appointed you at a specific time? **Check appointment card** | 1. Yes 2. No | |  |
| 419 | | Did you satisfy by the service given to you by the FP service providers during the insertion? | 1. Yes 2. No | |  |
| **Part V: Reasons for removal of Implanon use** | | | | | |
| **S.NO.** | **Questions** | | **Response** | | **Skip to/Remark** |
| 501 | When did you insert the implanon? (cross-check from card if available) | | DD/MM/YYYY  ____ /_______/_______ | |  |
| 502 | When did you remove implanon? (cross-check from card if available) | | DD/MM/YYYY  _____/______/____ E.C | |  |
| 503 | For how long did you utilize implanon? | | _______ months | |  |
| 504 | Reason for removal ( only for cases) | | 1. Side effects 2. Health concern 3. To get pregnant 4. Husband opposition 5. Religious opposition 6. Contraceptive failure 7. Inconvenience to use 8. Method shifts 9. Other/specify--------- | |  |
| 505 | Have you ever experienced unintended pregnancy after your implanon discontinued? | | 1. Yes 2. No | |  |

**Thank you for participating!!**

## **Hikaa Afaan Oromoo tiin**
