# Supplementary material for: Determinants of implanon discontinuation among women who ever used implanon in Shashemene district, west Arsi zone, Southern Ethiopia: unmatched case control study
Source: Contracept Reprod Med. 2023 Oct 3;8:46. doi: 10.1186/s40834-023-00248-6 (PMC10548583; doi:10.1186/s40834-023-00248-6)
Supplement: Supplementary file 2 — Supplementary Material 2 [file 40834_2023_248_MOESM2_ESM.docx]

STROBE Statement—checklist of items that was used for the study “Determinants of Implanon Discontinuation among Women Who Ever Used Implanon in Shashamane District, West Arsi Zone, Southern Ethiopia: Unmatched Case control study “

|  | Item No. | Recommendation | Page  No. | Relevant text from manuscript |
| --- | --- | --- | --- | --- |
| **Title and abstract** | 1 | (*a*) Indicate the study’s design with a commonly used term in the title or the abstract | 3 & 27 | Unmatched Case control study was done |
|  |  | (*b*) Provide in the abstract an informative and balanced summary of what was done and what was found | 27-41 | A Community based unmatched case-control study was carried out at Shashamane district among 264 (88 cases and 176 controls) women who were selected using systematic random sampling technique. Cases were women who discontinued Implanon before 3 years and controls were those who used Implanon for 3 complete years. A pretested, structured questionnaire with face to face interview was used. The collected data were entered using the Epi-Info 7.2.2 and analyzed using SPSS version 21.0. Bivariate and multivariable logistic regression analysis was performed to see the association between explanatory and outcome variables. Odds ratio with 95% confidence interval was used to show the strength of association and the significance was declared at α < 0.05.  **Result:** Women who were unable to read and write [AOR:3.09(1.20-8.00)], had less than four living children[AOR:2.47(1.20-5.08)], had no history of abortion [AOR:2.84(1.25-6.46)], new acceptors [AOR:2.14(1.02-4.49)], had counseled for less than fifteen minutes [AOR:2.47(1.29-4.70)], had no discussion with partner [AOR:2.88(1.42-5.84)] and experienced side effects [AOR:0.35(0.17-0.71)] were more likely to discontinue Implanon compared to their counter parts |
| Introduction | | | |  |
| Background/rationale | 2 | Explain the scientific background and rationale for the investigation being reported | 58-97 | Globally in 2019, 23 million of women were using implants which account 2% of all method users[[7](#_ENREF_7)]. In Sub-Saharan Africa, Implanon have been under-utilized despite its effectiveness and low cost[[8](#_ENREF_8)]. For instance, Ethiopian mini-demographic and health survey (EMDHS) 2019 reported that the implant users are about 9% and 7.4% in Ethiopia and Oromia among all method users respectively[[9](#_ENREF_9)].  In 2009, the Government of Ethiopia initiated the Implanon scale-up initiative, which facilitated greater Implanon access by allowing health extension workers (HEWs) to insert the implant. However, evidence from the 2016 Ethiopian demographic and health survey(EDHS) shows that 21.5% of implant episodes were discontinued at the end of 24 months[[10](#_ENREF_10)]. Studies in different parts of Ethiopia shows that implanon discontinuation remains high;16% in Ofla District of Tigray[[11](#_ENREF_11)], 36.9 % Andabat District of North West Ethiopia[[12](#_ENREF_12)], 23.4% in Dale District of Southern Ethiopia[[13](#_ENREF_13)], 34% in Kucha District Gamo Gofa Zone of Southern Ethiopia[[14](#_ENREF_14)],38.2% in Ambo town of central Ethiopia[[15](#_ENREF_15)], 46.5% in Debre Markos Town, Northwest Ethiopia[[16](#_ENREF_16)], 65% in Debre Tabor Town, Public Health Facilities, Northwest Ethiopia[[17](#_ENREF_17)], 38% in Mekelle City health institutions, Tigray, Ethiopia[[19](#_ENREF_19)] .  According to different studies conducted on Implanon discontinuation; educational status [[19](#_ENREF_19)], [[20](#_ENREF_20)], [[21](#_ENREF_21)], [[22](#_ENREF_22)]; age [[13](#_ENREF_13)], [[20](#_ENREF_20)]; income [[13](#_ENREF_13)]; counseling service [[12](#_ENREF_12)], [[13](#_ENREF_13)], [[15](#_ENREF_15)], [[16](#_ENREF_16)]; information on contraceptive methods[[13](#_ENREF_13)]; discussion with partner [[23](#_ENREF_23)]; decision making [[24](#_ENREF_24)]; mass counseling [[23](#_ENREF_23)]; follow up counseling [[12](#_ENREF_12)], [[13](#_ENREF_13)], [[14](#_ENREF_14)], [[25](#_ENREF_25)]; service satisfaction [[12](#_ENREF_12)],[[13](#_ENREF_13)], [[16](#_ENREF_16)]; side effects [[11](#_ENREF_11)], [[12](#_ENREF_12)], [[15](#_ENREF_15)], [[18](#_ENREF_18)], [[19](#_ENREF_19)], [[23](#_ENREF_23)], [[26](#_ENREF_26)], [[27](#_ENREF_27)]; desire for pregnancy [[28](#_ENREF_28)], [[29](#_ENREF_29)]; number of living children[[12](#_ENREF_12)], [[17](#_ENREF_17)], [[23](#_ENREF_23)]; history of pregnancy [[16](#_ENREF_16)] and history of previous abortion[[12](#_ENREF_12)] were associated with ID. Additionally, recently published studies also identified never used injectables or implants in the past, had experienced heavy/ prolonged bleeding, having medium monthly income, and residence as factors significantly associated with implanon discontinuation [[30-34](#_ENREF_30)]  Couple of systematic review and meta-analysis were conducted in Ethiopia and both of them reported prevalence of Implanon discontinuation to be more than 32% [[30](#_ENREF_30),[33](#_ENREF_33)]. However, they suffered from very high heterogeneity of more than 97% [[30](#_ENREF_30),[33](#_ENREF_33)]. This is because of the variation in population and the definition of implanon discontinuation. Furthermore, some of recent studies defined implanon discontinuation as removal of implanon by health professionals before 2.5 years of utilization[[32](#_ENREF_32)]. A follow up study done in Kinshasa, also followed the women for only 24 months[[27](#_ENREF_27)]. But, WHO recommends Implanon utilization for three complete years. Majority of the studies were also cross-sectional in nature and didn’t provide good evidence for cause effect relationship of exposure variables and Implanon discontinuation. Furthermore, some of the studies reported determinants of contraceptives discontinuation for long and permanent contraception not specific to Implanon. It is very important to undertake studies using relatively good Epidemiological study design and standard definitions. Therefore, this study was aimed to identify the determinants of implanon discontinuation among women in Shashamane district, Southern Ethiopia. |
| Objectives | 3 | State specific objectives, including any prespecified hypotheses | 94-96 | To identify the determinants of implanon discontinuation among women in Shashamane district, Southern Ethiopia |
| Methods | | | |  |
| Study design | 4 | Present key elements of study design early in the paper | 99-100 | A community based unmatched case control study was conducted among women women who ever used Implanon in Shashemene District. |
| Setting | 5 | Describe the setting, locations, and relevant dates, including periods of recruitment, exposure, follow-up, and data collection | 100-110 | It is located at 250 km South West to main city of Ethiopia, Addis Ababa. Shashamane district has 281,247 populations, 58,593 households and 61,874 women of reproductive age group residing in 37 rural kebeles. There are 7 health centers and 38 Health posts that provide health service for the community by 91 health extension workers and 144 health workers. In addition, there were 11 private clinics. All health centers and health posts provide implanon insertion and removal service. The prevalence of long-acting family planning utilization according to annual report in 2020/2021 was 17.4% and 26.8% in West Arsi Zone and Shashamane District respectively. Similarly, according to this report among women who have utilized long acting family planning in 2020/21; 23% of them in West Arsi Zone and 31% of them in Shashamane district have discontinued service before intended time[[35](#_ENREF_35)]. The study was conducted from April 12 to May 18, 2021 |
| Participants | 6 | (*a*) *Cohort study*—Give the eligibility criteria, and the sources and methods of selection of participants. Describe methods of follow-up  *Case-control study*—Give the eligibility criteria, and the sources and methods of case ascertainment and control selection. Give the rationale for the choice of cases and controls  *Cross-sectional study*—Give the eligibility criteria, and the sources and methods of selection of participants |  | All women of child bearing age group (15-49 years) who ever used Implanon in Shashamane district were the source population for this study. Cases are all women of child bearing age group in the selected kebeles who have discontinued their implanon before 3 years of insertion. Controls are all women of child bearing age group who used implanon for the complete 3 years in the selected kebeles. Women of reproductive age group, who had discontinued the implanon due to life threatening medical complications, method failure and who were critically ill at the time of the interview, were excluded from the study. |
|  |  | (*b*) *Cohort study*—For matched studies, give matching criteria and number of exposed and unexposed  *Case-control study*—For matched studies, give matching criteria and the number of controls per case |  | NA |
| Variables | 7 | Clearly define all outcomes, exposures, predictors, potential confounders, and effect modifiers. Give diagnostic criteria, if applicable | 147-163 | Implanon discontinuation was defined as a discontinuation of the use of Implanon before completion of three years [[36](#_ENREF_36)]. Card of mothers were used to check the date of insertion and removal of Implanon. In case of no card, women self-report was cross-checked with their file from health post or health facility  Independent variables were; age (<20, 20-24, 25-29, 30-34 & ≥ 35 years), place of residence (rural, semi-urban), marital status (married & others), ethnic group (Oromo, Sidama & others), religion (Muslim, Orthodox & Protestant), women occupation (housewife, farmer & others), partner occupation (farmer, merchant & others), women education(unable to read & write, able to read and write, primary & secondary), partner education (unable to read & write, able to read and write, primary, secondary & college & above), have children before insertion(yes, no), number of children (<4, ≥ 4), future intention to have children (yes, no), history of abortion (yes, no), ever used contraceptive before Implanon(yes, no), type of counseling (individual, with husband, mass), duration of counseling (< 15 minutes & ≥ 15 minutes), discussed with partner to use Implanon (yes, no), decision maker to use Implanon (self, husband & other), Implanon provider (health extension worker & health worker), side effect after insertion (yes, no), follow-up after insertion (yes, no), service satisfaction(yes, no) and unintended pregnancy after removal of Implanon (yes, no). |
| Data sources/ measurement | 8* | For each variable of interest, give sources of data and details of methods of assessment (measurement). Describe comparability of assessment methods if there is more than one group | *139-145* | The study used pretested structured questionnaire which was adapted from different literatures [[11](#_ENREF_11),[18](#_ENREF_18),[20](#_ENREF_20),[23](#_ENREF_23)]. The questionnaire contained: socio-economic and demographic characteristics of mothers, Obstetric history, past knowledge and utilization of contraceptive methods, counseling related factors and reasons for removal of Implanon (S1). Data collection was carried out by going home to home in selected kebeles of Shashamane district by five diploma nurses as data collectors and two-degree holder nurses as supervisors. The data were collected from women of child bearing age group by face to face interview. |
| Bias | 9 | Describe any efforts to address potential sources of bias | 319-326 | Social desirability was possible because of self-report of the time period of ID. The possibility was minimized by cross-checking with the family card of the mother and also from health facility. Selection bias was minimized since it is a community based study. Both cases and control s come from the same source population. To reduce recall bias target women who had been discontinued implanon in the last three years prior to the data collection were used. This study must be interpreted considering the above limitations. |
| Study size | 10 | Explain how the study size was arrived at | 120-124 | The sample size was calculated using two population proportion formula for unmatched case control study by Epi info version 7.2.2 with the assumption of level of significance 5%, power 80%, case to control ratio (1:2), odd ratio 2.3 and percent of controls with previous history of abortion 28.6 [[20](#_ENREF_20)] and finally considering 10% non-response rate, the final sample size was estimated to be 264 (88 cases and176 controls) |

Continued on next page

| Quantitative variables | 11 | Explain how quantitative variables were handled in the analyses. If applicable, describe which groupings were chosen and why |  | Quantitative variables were categorized. and used for analysis ; age (<20, 20-24, 25-29, 30-34 & ≥ 35 years); number of children (<4, ≥ 4), |
| --- | --- | --- | --- | --- |
| Statistical methods | 12 | (*a*) Describe all statistical methods, including those used to control for confounding | 177-187 | The data were checked for completeness and entered into Epi info version 7.2.2 and imported to statistical package for social science (SPSS) version 25.0 for analysis. Descriptive statistics including frequency and proportion were computed to describe the data. Bi-variate and multivariable logistic regression analysis was performed to see the association between outcome and explanatory variables. Variables that have P-value < 0.2 in the bivariate analysis were entered in to multivariable logistic regression model. Finally, multivariable logistic regression analysis was done to identify factors associated with Implanon discontinuation. During multivariable analysis, model fitness was checked using the Hosmer and Lemeshow`s goodness of fitness test (p = .412). Adjusted odds ratio along with 95% Confidence Interval (CI) was used to assess the strength of association. Level of statistical significance was declared at p-value less than 0.05. |
|  |  | (*b*) Describe any methods used to examine subgroups and interactions |  |  |
|  |  | (*c*) Explain how missing data were addressed |  |  |
|  |  | (*d*) *Cohort study*—If applicable, explain how loss to follow-up was addressed  *Case-control study*—If applicable, explain how matching of cases and controls was addressed  *Cross-sectional study*—If applicable, describe analytical methods taking account of sampling strategy |  |  |
|  |  | (*e*) Describe any sensitivity analyses |  |  |
| Results | | | | |
| Participants | 13* | (a) Report numbers of individuals at each stage of study—eg numbers potentially eligible, examined for eligibility, confirmed eligible, included in the study, completing follow-up, and analysed | 199-200 | A total of 264 women (88 cases and 176 controls) were participated in the study giving a response rate of 100 % for both cases and controls. |
|  |  | (b) Give reasons for non-participation at each stage |  |  |
|  |  | (c) Consider use of a flow diagram |  |  |
| Descriptive data | 14* | (a) Give characteristics of study participants (eg demographic, clinical, social) and information on exposures and potential confounders | 200-204 | The mean age of respondents was 28.23 (±5.465) years: 27.27 (±5.387) for cases and 28.70 (±5.525) years for controls. Most women, 83 (94.3%) of cases and 173 (98.3%) of controls were married. About one third 30 (34.1%) of cases and 69 (39.2 %) controls have attained primary school. About 80 (90.9%) of cases and 151(85.8%) controls were housewives in employment |
|  |  | (b) Indicate number of participants with missing data for each variable of interest |  |  |
|  |  | (c) *Cohort study*—Summarise follow-up time (eg, average and total amount) |  |  |
| Outcome data | 15* | *Cohort study*—Report numbers of outcome events or summary measures over time |  |  |
|  |  | *Case-control study—*Report numbers in each exposure category, or summary measures of exposure | *209-232* | According to the findings of this study, majority of the respondents, 75 (85.2%) cases and 160 (90.9%) controls obtained information about contraceptive methods from health extension workers. Most of the respondents, 54(61.4%) cases and 141(80.1%) controls had used other contraceptives before taking Implanon. The most common contraceptive method ever used before Implanon was Injectable; 34(38.6%) for case and 93(52.8%) for controls  This study found that 81(92.0%) cases and 167(94.9%) controls had children before insertion of the Implanon. This study also showed that 45 (51.1%) of cases and 71 (40.3%) controls had < 4 children. Majority of the respondents, 82(93.2%) cases and 161(91.5%) controls had future intention to have children. Among the total study participants, 125 (71.0%) cases and 75 (85.2%) controls had no history of abortion.  About 70 (79.5%) cases and 140(79.5 %%) controls had an individual pre-counseling service and 60(68.2%) cases and 83(47.2%) of controls were counseled for less than fifteen minutes respectively. Among those who have used Implanon, 54 (61.4%) cases and 140 (79.5%) controls had discussion with their partners. Majority of the women, 78(88.6%) of cases and 147(83.5%) of controls got implanon insertion service by health extension workers. According to this study, 59(67%) of cases and 54(30.7%) of controls were experienced side effect, and most of the respondents 59(67.0%) cases and 137(77.8%) controls had follow-up after insertion of implanon. From 88 cases that discontinued their implanon before 3 years, 14(15.9%) said that their pregnancy was unintentional |
|  |  | *Cross-sectional study—*Report numbers of outcome events or summary measures |  |  |
| Main results | 16 | (*a*) Give unadjusted estimates and, if applicable, confounder-adjusted estimates and their precision (eg, 95% confidence interval). Make clear which confounders were adjusted for and why they were included | 236-259 | In the bivariate logistic regression, age, maternal educational status, number of children, history of abortion, ever-used contraceptive before, duration of counseling, discussion with partner, side effects, menstrual bleeding pattern after insertion, follow-up appointment were found to be candidate variables for multivariable analysis at p-value of < 0.2.  After adjusting variables in a multivariable logistic regression model, women`s educational status, number living children less than four, history of abortion, ever use of any contraceptive before insertion of implanon, duration of counseling, discussion with their partner and experiencing side effects after insertion were determinants of Implanon discontinuation.  Women who can`t read and write were 3.09 times more likely to discontinue their implanon use as compared to women who attended secondary school, [AOR= 3.09(1.20- 8.00)]. The study also showed that women who had living children less than 4 were 2.47 times more likely to discontinue their implanon use when compared to those who had four and more children at time of insertion, [AOR = 2.47(1.20-5.08)]. The odds of implanon discontinuation was 2.84 times higher among women who had no history of abortion when compared to their counterpart, [AOR=2.84(1.25-6.46)]. According to this study, the odds of implanon discontinuation among new acceptors were 2.14 times than those who had ever used any contraceptive before [AOR = 2.14(1.02-4.49)]. The analysis showed that women who had counseled for less than fifteen minutes were 2.47 times more likely discontinue their implanon use when compared to those who had counseled for fifteen minutes and more [AOR = 2.47(1.29-4.70)]. The odds of implanon discontinuation among women who had not discussed with their partners were 2.88 times than those who had discussed with their partners [AOR = 2.88(1.42-5.84)]. This study also showed that women who had no side effects after insertion of implanon were 65 % less likely to discontinue their implanon use as compared to women who had experienced side effects after insertion of implanon [AOR =0.35(0.17-0.71)] |
|  |  | (*b*) Report category boundaries when continuous variables were categorized |  | Age (<20, 20-24, 25-29, 30-34 & ≥ 35 years); number of children (<4, ≥ 4) |
|  |  | (*c*) If relevant, consider translating estimates of relative risk into absolute risk for a meaningful time period |  |  |

Continued on next page

| Other analyses | 17 | Report other analyses done—eg analyses of subgroups and interactions, and sensitivity analyses |  |  |
| --- | --- | --- | --- | --- |
| Discussion | | | | |
| Key results | 18 | Summarise key results with reference to study objectives |  | The study aimed to identify determinants of ID among women who ever used Implanon in Shashemene district. This study found that that those women who were unable to read and write, had less than four living children, had no history of abortion, new acceptors, had counseled for less than fifteen minutes, had no discussion with partner and experienced side effects were more likely to discontinue Implanon compared to their counter parts. |
| Limitations | 19 | Discuss limitations of the study, taking into account sources of potential bias or imprecision. Discuss both direction and magnitude of any potential bias | 320-326 | Since data collection method was face-to-face interview, there might be possibility of social desirability bias. Social desirability was also possible because of self-report of the time period of ID. Social desirability bias may lead to misclassification of cases and controls in our study. The possibility was minimized by cross-checking with the family card of the mother and also from health facility. Selection bias was minimized since it is a community based study. Both cases and control s come from the same source population. Because of the nature of the design, there might be recall bias. To reduce recall bias target women who had been discontinued implanon in the last three years prior to the data collection were used. This study must be interpreted considering the above limitations. |
| Interpretation | 20 | Give a cautious overall interpretation of results considering objectives, limitations, multiplicity of analyses, results from similar studies, and other relevant evidence | 326 -329 | Despite the limitations listed above, this study provides valuable evidence about determinants of Implanon discontinuation which could be utilized by program planners and policy makers to further strengthen the implant use among women in Shashmene in particular and in Ethiopia in General . This finding implies that low level of education; inadequate counseling and poor communication among couples were major problems that needs immediate intervention. |
| Generalisability | 21 | Discuss the generalisability (external validity) of the study results |  | This study could be generalized to Shashemene district and west arsi zone since they are in similar socioeconomic level. |
| Other information | |  | | |
| Funding | 22 | Give the source of funding and the role of the funders for the present study and, if applicable, for the original study on which the present article is based | 36-361 | No fund was obtained for this research. All costs of data collection and analysis were covered by the authors. |

*Give information separately for cases and controls in case-control studies and, if applicable, for exposed and unexposed groups in cohort and cross-sectional studies.

**Note:** An Explanation and Elaboration article discusses each checklist item and gives methodological background and published examples of transparent reporting. The STROBE checklist is best used in conjunction with this article (freely available on the Web sites of PLoS Medicine at http://www.plosmedicine.org/, Annals of Internal Medicine at http://www.annals.org/, and Epidemiology at http://www.epidem.com/). Information on the STROBE Initiative is available at www.strobe-statement.org.
