# Supplementary material for: Determinants of implanon discontinuation among women who ever used implanon in Shashemene district, west Arsi zone, Southern Ethiopia: unmatched case control study
Source: Contracept Reprod Med. 2023 Oct 3;8:46. doi: 10.1186/s40834-023-00248-6 (PMC10548583; doi:10.1186/s40834-023-00248-6)
Supplement: Supplementary file 3 — Supplementary Material 3 [file 40834_2023_248_MOESM3_ESM.docx]

Cover letter ID

Our study is original article that contributes to scientific literatures by identifying determinants of Implanon Discontinuation among women in Shashemene District of Southern Ethiopia. We have used literatures published on reputable journals as a reference for this study. Our manuscript is not under consideration for publication in any other journal currently. Any academic editor who is expert on our study area may handle our manuscript. We don’t oppose any reviewer.
